# Selection for territory acquisition is modulated by social network structure in a wild songbird

**DOI:** 10.1111/jeb.12587

**Published:** 2015-02-06

**Authors:** D R Farine, B C Sheldon

**Affiliations:** * Department of Zoology, Edward Grey Institute of Field Ornithology, University of OxfordOxford, UK; † Smithsonian Tropical Research InstituteAncon, Panama; ‡ Department of Anthropology, University of CaliforniaDavis, CA, USA

**Keywords:** fission–fusion dynamics, group-living, natural selection, *Paridae*, phenotypic composition, population structure, social network analysis, social selection

## Abstract

The social environment may be a key mediator of selection that operates on animals. In many cases, individuals may experience selection not only as a function of their phenotype, but also as a function of the interaction between their phenotype and the phenotypes of the conspecifics they associate with. For example, when animals settle after dispersal, individuals may benefit from arriving early, but, in many cases, these benefits will be affected by the arrival times of other individuals in their local environment. We integrated a recently described method for calculating assortativity on weighted networks, which is the correlation between an individual's phenotype and that of its associates, into an existing framework for measuring the magnitude of social selection operating on phenotypes. We applied this approach to large-scale data on social network structure and the timing of arrival into the breeding area over three years. We found that late-arriving individuals had a reduced probability of breeding. However, the probability of breeding was also influenced by individuals’ social networks. Associating with late-arriving conspecifics increased the probability of successfully acquiring a breeding territory. Hence, social selection could offset the effects of nonsocial selection. Given parallel theoretical developments of the importance of local network structure on population processes, and increasing data being collected on social networks in free-living populations, the integration of these concepts could yield significant insights into social evolution.

## Introduction

One of the fundamental motivations in the study of evolution in natural populations is to understand the causes of relationships between phenotypic trait distributions and fitness, quantified as selection (Endler, 1986; Wade & Kalisz, 1990; Kingsolver *et al*., 2001). The environment is a key determinant of an individual's fitness, and indeed, understanding the causes of variation in selection is fundamentally an ecological challenge (Wade & Kalisz, 1990; Moore *et al*., 1997; MacColl, 2011). Although natural selection is well known to favour particular values of individual traits, including social phenotype (e.g. position in a social network, see Formica *et al*., 2012; Wey *et al*., 2013), less is known about the role of social selection in mediating individual fitness (Wolf *et al*., 1999; Formica *et al*., 2011). In many species that exhibit social behaviour, the environmental causes of selection can include selection that is mediated by the interaction between a focal individual's phenotype and the phenotype of their associates (West-Eberhard, 1979; Wolf *et al*., 1999; McGlothlin *et al*., 2010). In this way, social interactions can shape evolution by generating variance in individual fitness when the distribution of phenotypes in social groups is nonrandomly drawn from the wider population. This suggests that phenotypic assortment – the grouping or avoidance of like individuals – should be a critical parameter under investigation in social species.

Previous studies aiming to understand the evolutionary consequences of social behaviour in animals have generally focused on the importance of individual roles in determining fitness (Szekely *et al*., 2010; Westneat & Fox, 2010) or the selection on phenotypes imposed by environmental niche space (MacColl, 2011). However, it may be difficult to determine whether selection acts directly on the match of phenotypes to their environment (natural selection) or is influenced by the relative state of phenotypes compared to competitors or social partners (social selection). Understanding the structure of social interactions in animal populations can tell us more about the social environment that individuals experience, thus better informing social selection analyses. For example, Price *et al*.'s classic study of drought-induced natural selection on beak size in *Geospiza fortis* (Price *et al*., 1984) would seem a prime candidate for selection imposed directly by the environment. However, as Grant & Grant (2006) subsequently showed, in the presence of a larger competitor species the direction of selection on bill size during droughts changed, suggesting that interactions with other individuals could play an important role in determining selection on individual phenotypes. In some cases, the form of selection on phenotypes is likely to depend on relative measures, which may be determined by local effects (e.g. who an individual interacts with – their social environment). An example of a localized measure is social dominance, where benefits relate to having a higher relative rank rather than absolute dominance. In other situations, it may pay to associate with the same phenotype, for example groups of morphologically identical individuals can gain benefits from predator confusion over and above the benefits of predator dilution (Landeau & Terborgh, 1986). In both these cases, selection on the phenotypic trait of an individual is only under selection in the context of their social environment (how that trait maps to the traits of those it interacts with).

Wolf *et al*. (1999) proposed that the total effect of selection could be partitioned between the selection gradient operating on the individual's phenotype (natural, or nonsocial, selection) and selection arising from phenotypic assortment (social selection). They extended the measure of selection gradient proposed by Lande & Arnold (1983), using partial regression of the relative individual and social trait contributions to the observed fitness. Formica *et al*. (2011) applied this framework to forked fungus beetles (*Bolitotherus cornutus*), demonstrating that large males gained greater copulation success when associating with small males than expected from their size alone. In this case, Formica *et al*. (2011) proposed that the total effect of selection *s* could be calculated for a single trait using:


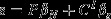
(1)

where *P* is the phenotypic variance of the trait in the observed individuals (where *P* = 1 when traits are standardized) and *C*^*I*^ is the covariance of interacting phenotypes (i.e. whether phenotypes interact nonrandomly or are assorted). The gradients of nonsocial selection(*β*_*N*_) and social selection (*β*_*s*_) are estimated by partial regression of relative fitness for each individual on its trait and the phenotypic traits of its associates (the equation for estimating these gradients is provided in the methods). This formulation suggests that social and natural selection gradients can operate simultaneously on individuals and also that individuals may be able to modify the strength of natural selection through their choice of social niche (Eldakar *et al*., 2009; Formica & Tuttle, 2009).

To estimate the relative gradients of natural and social selection, we need to accurately measure the phenotypic covariance between interacting phenotypes. Social network analysis is a quantitative approach that is typically used to capture the emergent population-level properties of repeated interactions between individuals (Whitehead, 1997, 2008; Krause *et al*., 2007; Croft *et al*., 2008); it has been suggested as an accessible way of estimating the evolutionary consequences of social processes (Krause *et al*., 2007; Croft *et al*., 2008; Wey *et al*., 2008; Farine *et al*., 2012). Importantly, network analysis provides a set of standard methods for collecting data and estimating rates of interactions between individuals in populations (Farine, in press). A number of studies have linked individual fitness to individual position in a social network. For example, males with more associates had greater copulation success in forked fungus beetles (Formica *et al*., 2012), whereas female degus (*Octodon degus*) with more varied associations had lower reproductive success (Wey *et al*., 2013). However, these studies have not considered how selection may be further influenced by the phenotypes of individuals connected in a social network, that is the effects of social selection. In the current study, we use assortative mixing, which seems ideally suited to quantify the covariance matrix of interacting phenotypes *C*^*I*^, in order to investigate whether the relative phenotypes of interacting individuals in a social network can influence selection through social selection.

In this study, we investigate the effect of social structure, in terms of arrival time, on territory acquisition in a wild bird population. In many species, individuals disperse from their natal territories shortly after reaching independence and often have a prolonged period before having the opportunity to breed themselves. By settling in a location early in this phase, individuals may gain advantages over competitors by acquiring more information or familiarity about their environment (Forero *et al*., 2002; Nocera *et al*., 2006; Clobert *et al*., 2009; Nocera & Betts, 2010), gaining social dominance (Koivula *et al*., 1993) or accessing the best breeding sites (Lens & Dhondt, 1994). However, this advantage may be dependent on the individual's arrival relative to the arrivals of others in the locations in which they settle. Therefore, the outcomes of dispersal are likely to be influenced by social interactions when social dominance is strongly linked to residency time. We use a large-scale study using winter social networks to determine whether the population is assorted by arrival time, and the consequences of this on the acquisition of breeding territories in a population that is known to have a surplus of birds that fail to breed (Krebs, 1971). We expected that birds that arrived early would have a higher chance of gaining a breeding territory, benefiting from residence-related dominance (Sandell & Smith, 1991), but that late-arriving birds might be able to reduce the fitness consequences of late arrival if they associated with even later arrivals.

## Materials and methods

### Study system and estimation of fitness

The study was conducted on the population of great tits (*Parus major*) at Wytham Woods, Oxford (51**°**46′N, 01**°**20′W), starting in May 2011 and running until July 2014. Wytham Woods is a 385 ha area of broadleaf deciduous woodland and is surrounded by farmland. As part of long-term monitoring of this population, all breeding attempts in an array of over 1000 nest boxes are recorded (Charmantier *et al*., 2008). Pairs of great tits defend territories over the breeding season, during which the majority of breeding adult birds were caught. These were fitted with uniquely coded metal rings supplied by the BTO and a passive integrated transponder (PIT) tag, enabling automated detection by radio frequency identification (RFID) antennae (see below for details of data collection protocol for these data). Every surviving nestling was also fitted with both a metal ring and a PIT tag at 15 days old. To capture, ring and tag birds that immigrated into Wytham Woods, extensive mist netting was conducted during the autumns of 2011–2013. Birds were also regularly mist-netted in villages and farms immediately surrounding Wytham Woods. We limited our analyses of selection to birds breeding in their first year (herein ‘juveniles’) to avoid potential effects of dominance due to prior breeding. Because an analysis incorporating all birds (i.e. both those tagged as breeding birds and nestlings) could be biased by immigrant juveniles being caught after having dispersed into Wytham Woods, we repeated our analyses for the restricted subset of birds that already were tagged prior to the start of winter (i.e. those born in Wytham Woods). In all cases, the social networks, which we used to quantify patterns of association, consisted of all individuals in the population (both adult and juvenile).

We were interested in territory acquisition as a fitness trait. Given the high mortality rates of small passerine birds, such as the great tit (with an average annual survival rate of 0.48; Bouwhuis *et al*., 2012), selection strongly favours breeding as early as possible. Hence, failure to breed in the first year of life has a major impact on lifetime fitness. Individuals were detected at breeding nest boxes and were given a value of 1 if they were detected breeding in the spring and 0 if they were not detected; that is, birds given a value of 0 were assumed to have been unsuccessful in acquiring a territory within Wytham Woods, remaining in the population as floaters (Krebs, 1971). To capture the identity of both males and females, we identified parents after chicks hatched by fitting a PIT-tag-detecting faceplate to every known great tit nest when chicks were between 2 and 5 days old. The normal faceplate on each nest box is detachable, and these were temporarily replaced with a customized faceplate fitted with a built-in RFID antenna (Dorset ID) for up to one hour. The identification of parents already fitted with a PIT tag (approximately 90% of all breeding birds, Aplin *et al*., 2013) was determined as they entered the nest box to feed or brood chicks.

### Arrival time

The territorial behaviour of great tits dissolves post-breeding, and the population exhibits fission–fusion dynamics (Farine *et al*., 2012; Aplin *et al*., 2013, in press). Generally, approximately half of the breeders in Wytham Woods are born outside of the study area (Verhulst *et al*., 1997). Recent multimodal mark–recapture modelling has determined that these birds arrive in two waves, the first in mid-autumn and another in late winter (Matechou *et al*., in press). In addition to immigration, up to 90% of locally born birds can leave the wood during summer, timing their return during these same two waves (Gibb, 1954; Matechou *et al*., in press).

To estimate the arrival time of individuals into Wytham Woods, and to identify their social affiliates, we used a stratified grid (approximately 250 m spacing) of 65 automated feeding stations fitted with RFID antenna and filled with sunflower seed. Social data were collected from the first week of December to the first week of March each winter. Each week, feeding stations were programmed to automatically open for the same two days each week, thereby providing a snapshot of the population structure at a fixed (weekly) interval by detecting the unique tags fitted to each individual as they visit the feeders.

From these data, we extracted the earliest date that each individual great tit was detected. Birds were then assigned the week number (where a value of 1 was given to birds detected in the first week) as the delay in arrival time into Wytham woods. Arrival dates measured using this approach were consistent with the timings estimated using a novel multimodal mark–recapture analysis conducted on the first year of data (Matechou *et al*., in press). Arrival times were then standardized to zero mean and unit variance to report standardized values of selection.

### Inferring the social network

Associations between individuals were inferred from their co-occurrence at the automated feeding stations. We used a recently developed method implementing Gaussian mixture models (Psorakis *et al*., 2012) that detects nonrandom bursts of activity, defining each burst as a gathering event. This is a probabilistic model that is used to detect the presence of bursts of arrivals at a feeder within a continuous data stream (time-stamped detections of PIT-tagged birds). Biologically, this method enabled us to detect temporally focused ‘waves’ of foraging birds forming flocks rather than using a fixed window of time for defining co-visitations. Using simulations, we found that this method best detected known social structure in our data (Psorakis *et al*. in revision). Thus, our stratified grid captures the spatial distribution of birds that underpins the population structure (most birds only visit a single feeder all winter Aplin *et al*., 2013), whereas using machine-learning to identify flocks captures the patterns of attraction and avoidance that determine local social structure (Farine, 2013b).

We used the gambit of the group approach (Whitehead, 2008; Franks *et al*., 2010) where individuals were assigned to foraging flocks using the Gaussian mixture model. From repeated co-observations of individuals visiting the same feeder at the same time (thus in the same flock), we calculated dyadic association strength (the simple ratio index, Whitehead, 2008) using the asnipe package in r (Farine, 2013a). Thus, network edges, or associations, represent the probability of two individuals co-occurring in a flock and ranges from 0 (never observed together) and 1 (always observed together), and capturing the spatiotemporal overlap in the distribution of individuals across the study area (and thus competition for breeding territories).

### Comparing winter social networks to breeding locations

We took two different approaches to determine whether individuals that associated together during the winter were also likely to be competing for the same nestboxes. First, we compared the Euclidian distance between the breeding locations of birds with a connection (those that were observed together at least once) to the distance between breeding locations of birds that were not connected (never observed together) in the social network. We performed this comparison within each year separately using a *t*-test. Second, we evaluated whether birds with stronger connections also bred closer to each other by fitting a linear model of the Euclidian distance as a function of the edge weight (for each year separately). To test whether this effect was stronger than expected, given the location of nestboxes in Wytham Woods, we compared the slope of the linear model to the slope of the same model applied to data sets made up of the same social network but where the assignment of boxes was randomly allocated from all available nestboxes (repeating this 1000 times). A slope for the model based on the observed data that is more negative than the slopes from the randomized data suggests that associates bred closer to each other than expected by chance.

### Estimating social selection in social networks

When applied to a single trait, the interaction covariance *C*^*I*^ in eqn 1 (see Introduction) can be defined as the Pearson product-moment correlation coefficient of a focal individual's phenotype and that of its associates (Formica *et al*., 2011). In the context of social networks, this is the same measure as the assortativity coefficient, a measure of similarity or dissimilarity in the trait of associates in a social network using the Pearson correlation coefficient (Newman, 2003). Thus, we can calculate the interaction covariance *C*^*I*^ using the weighted assortment in arrival time 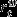 which is given by (Farine, 2014):


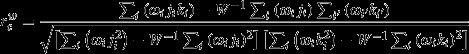
(2)

where *j*_*i*_ and *k*_*i*_ are the arrival times of individuals that edge *i* leads into and out of, respectively, *ω*_*i*_ is the weight of edge *i*, and *W* is the sum of all edge weights. In this equation, if the network is assorted, then 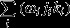 becomes much larger than 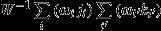, leading to positive values of 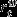. In contrast, if the network is disassorted, then 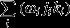 is smaller than 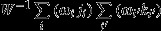, resulting in negative values of 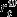. The denominator of the equation serves to scale 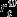 between −1 and 1. To test whether the assortment was significant, we compared the assortment value in the observed network to the assortment value calculated on 1000 randomizations of the network (data randomizations following Farine, 2014). This test is based on repeatedly swapping observations of individuals in the network whereas keeping the number of observations and group sizes constant.

The value of *P* in eqn 1 is the variance of the arrival time of individuals, which we normalized to 1 with unit variance. The values of *P* and *C*^*I*^ are then used to scale the gradients of selection, given by coefficients *β*_*N*_ and *β*_*s*_ in eqn 1, to calculate the total effect of selection. These coefficients represent the nonsocial and social gradients of selection, which we estimated using a linear multiple regression on the fitness of each individual using (from Wolf *et al*., 1999):


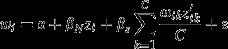
(3)

where fitness *w*_*i*_ represents whether the individual acquired a territory, *α* is a constant, *z*_*i*_ denotes the individual's arrival time, 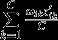 is the individual's social environment (the weighted mean arrival time of its associates, calculated by multiplying the arrival time 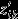 of all other individuals *k* by the edge weight *ω*_*ik*_ – where nonassociating individuals have an edge weight of 0 – divided by the weighted degree *C* which is the sum of all edges connected to individual *i*), and *ε* is an error term whose variance is to be minimized. Given that the fitness measure was binary (0 or 1), we specified a binomial error distribution in the regression model. If *β*_*N*_ is negative, then there is selection against late arrival (individuals that arrived later had a lower probability of acquiring a territory). If *β*_*s*_ is positive, then individuals gain a positive effect of social selection by having associated with later arrivals.

### Estimating brood-level effects

We tested for brood-level effects on arrival time, which would suggest that social selection might be a cascading effect from natal effects. We fitted a slope-only generalized linear mixed model of the complete data (3 years), with arrival time as the dependent variable, and the brood identifier (breeding box ID) and year as random effects. This enabled us to estimate the amount of variance in arrival time that was explained by an individual's natal brood.

### Estimating competition for breeding territories

To investigate the role of competition as a mechanism of selection for early arrival, we calculated the ratio of conspecific competitors to available nest boxes in each individual's wintering range. The number of conspecific competitors was calculated based on the proportion of time that individuals spent in each location, summed by location, to generate an estimated total population size at each feeder site. We assigned each great tit nest box (*N* = 1077) to the nearest feeder site (*N* = 65) and estimated local competition by dividing the local population size (the number of individuals times the proportion of time they spent at that location) by the number of nest boxes used. For each individual, we then calculated the weighted average competition from their foraging range based on the amount of time they spent at each location.

## Results

### Arrivals into Wytham Woods

Over the three years, 62.8% of individual birds were present when we began collecting data (the early arrivals). The mean arrival time for late arrivals was 7.2 weeks (median = 7) after the start of data collection (i.e. late January). In total, 11% of all great tits arrived into Wytham Woods during the month of February, which represents the second wave of incoming birds (c.f Matechou *et al*., in press). These patterns were similar when considering locally born birds alone (66.9% were present at the start of data collection and 7.1% arrived during the month of February).

### Social network

The social networks were sparse in all three winters (network densities were 0.08, 0.06 and 0.06, respectively), but networks in all years formed a single fully connected component. We detected 3 347 038 feeder visits by 1053 individual great tits over 3 months in the 2011/2012 winter. From these, the Gaussian mixture model identified 73 737 unique groups from which we formed the social network. Of these great tits, 520 (49.4%) were first-year juveniles. Over the same period in 2012/2013, we detected 2 574 698 visits by 729 individuals (152 or 20.9% of which were juveniles), forming 68 057 flocks. In 2013/2014, we detected 2 572 441 visits by 816 great tits (411 juveniles, or 50.3%), forming 70 447 groups. The difference in age structure in the second winter results almost wholly from differences in nestling productivity in the 2012 breeding season which was the lowest recorded in the 53 years of this study since 1962.

Individuals were significantly assorted by their arrival date in the social networks each year. In 2012, the assortativity coefficient 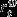 (± SE) was 0.288 ± 0.003 overall and 0.359 ± 0.005 in juveniles. In 2013, the assortativity coefficient was slightly lower at 0.188 ± 0.004 overall and 0.214 ± 0.018 in juveniles. In 2014, the assortativity coefficient was 0.352 ± 0.003 in adults and 0.470 ± 0.008 in juveniles. In all three years, the assortativity coefficient was higher in juveniles than in adults. The networks were all significantly more strongly assorted than expected by chance (*P* < 0.001 in all cases, estimated using data permutations).

### Associations in the social network location of breeding

In all three years, individuals associated in the social network bred significantly closer to each other than individuals not associated (Year 1: associates = 1309 m, nonassociates = 1625 m, *t* = −45.76, *P* < 0.001; Year 2: associates = 1230 m, nonassociates = 1536 m, *t* = −27.53, *P* < 0.001; and Year 3: associates = 405 m, nonassociates = 1668 m, *t* = −270.66, *P* < 0.001). Further, individuals with stronger edge values (those that were observed more often together) also bred closer to each other (Year 1: coef ± SE = −744 ± 122, *t* = −6.07, P_rand_ < 0.001; Year 2: coef ± SE = −1373 ± 145, *t* = −9.41, *P*_rand_ < 0.001; and Year 3: coef ± SE = −2383 ± 68, *t* = −35.2, *P*_rand_ < 0.001; note *P*_rand_ is calculated using randomizations, see Methods). Thus, individuals were likely to compete disproportionately more for territories with close social associates than with nonassociates.

### Winter social networks and territory acquisition

We identified 83% of all possible breeding birds (1918 identities recorded from a maximum of 1151 breeding attempts) over the three years. However, this figure is likely to be an underestimate of the proportion of breeding great tits identified as early nest failure (before parents are identified) often results in repeat breeding at the same, or an adjacent, site (DRF, BCS – pers. obs).

Across all three years, we found strong support that both non-social and social selection were operating (Table 1). Of all juveniles, 32–40% were detected having successfully acquired a territory each year. Selection for arrival date was negative in all years (Year 1: *Pβ*_*N*_ = −0.776, Year 2: *Pβ*_*N*_ = −0.220, and Year 3: *Pβ*_*N*_ = −0.325; *P =* 1 as it is normalized to unit variance). Late arrivals typically had a lower likelihood of acquiring a territory. These estimates of nonsocial selection on arrival date are consistent with work showing a link between arrival date and subsequent breeding derived from a novel multimodal mark-and-recapture analysis in the same population and applied to the first year of our study (Matechou *et al*., in press). The latter technique models the observation process, so we are confident that our conclusions are robust.

**Table 1:** Social and nonsocial selection gradients of winter dispersal phenotype on spring territory establishment for all first-year great tits in each year

Year	Juveniles	On territory	Type of selection	Trait	*β*^(standardised)^	SE	*P*
2011/12	520	174	Nonsocial	Arrival date	−0.776	0.148	< 0.001
			Social	Weighted arrival date of associates	1.152	0.307	< 0.001
2012/13	152	48	Nonsocial	Arrival date	−0.220	0.187	0.240
			Social	Weighted arrival date of associates	1.021	0.610	0.094
2013/14	411	164	Nonsocial	Arrival date	−0.325	0.100	0.001
			Social	Weighted arrival date of associates	0.463	0.212	0.029

Social network analysis was used to capture the overall structure of the population and the social environment experienced by each bird. Using the assortativity coefficient to estimate population-level structure (where *C*^*I*^ *=* 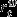 by fitting eqn 2 into eqn 1) suggested that positive assortment for arrival time between social associates led to positive counteracting selection in all three years (Year 1: *C*^*I*^ *β*_*s*_ = 0.332, Year 2: *C*^*I*^ *β*_*s*_ = 0.192 and Year 3: *C*^*I*^ *β*_*s*_ = 0.162). Thus, associating with conspecifics that arrived into the study area late resulted in a positive benefit on the likelihood of acquiring a territory.

**Fig 1:**
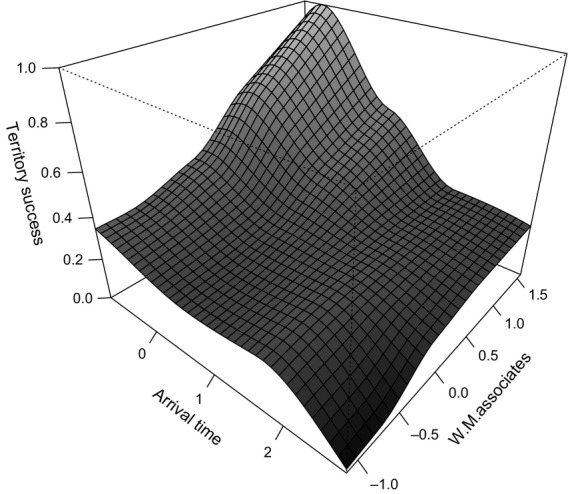
Selection gradient of the probability of acquiring a breeding territory as a function of the delay in arrival time (normalized to units of standard deviations) and the weighted mean of associate's arrival time (W.M. Associates, in standard deviations). Data are combined for all three years.

When nonsocial and social selection are combined (using eqn 1, Formica *et al*., 2011), we found that overall selection was consistently driven by nonsocial selection, but that it was substantially weakened by positive effects of social selection (Year 1: *s* = −0.444, Year 2: *s* = −0.028 and Year 3: *s* = −0.167). Arriving late into Wytham Woods was selected against in all three years of the study (Fig. 1), but the strength of this selection was strongly dependent on the varying degrees of natural selection and social selection.

Results remained consistent when we considered only locally born birds, which were all fitted with PIT tags as nestlings (Table 2). In all three years, the nonsocial components of selection were similar to the estimates for the full complement of juveniles (Year 1: *Pβ*_*N*_ = −0.620, Year 2: *Pβ*_*N*_ = −0.231 and Year 3: *Pβ*_*N*_ = −0.460). However, the effects of social selection were much stronger in these birds (Year 1: *C*^*I*^ *β*_*s*_ = 0.537, Year 2: *C*^*I*^ *β*_*s*_ = 0.506 and Year 3: *C*^*I*^ *β*_*s*_ = 0.200), presumably because they were more likely to be among the longest resident in their social groups. As a result, overall selection on locally born juveniles was generally lower and fluctuated between positive and negative values (Year 1: *s* = −0.083, Year 2: *s* = 0.275 and Year 3: *s* = −0.260).

**Table 2:** Social and nonsocial selection gradients of winter dispersal phenotype on spring territory establishment for all first-year great tits restricted to locally born great tits that were fitted with PIT tags as nestlings

Year	Juveniles	On territory	Type of selection	Trait	*β*^(standardised)^	SE	*P*
2011/12	283	105	Nonsocial	Arrival date	−0.620	0.191	0.001
			Social	Weighted arrival date of associates	1.508	0.443	< 0.001
2012/13	83	30	Nonsocial	Arrival date	−0.231	0.234	0.813
			Social	Weighted arrival date of associates	2.363	1.015	0.020
2013/14	138	110	Nonsocial	Arrival date	−0.460	0.100	< 0.001
			Social	Weighted arrival date of associates	0.424	0.276	0.125

### Brood-level effects

To estimate whether the social selection we observed could be attributed to carry-over natal effects, we constructed a model with arrival time as a dependent variable, and both brood ID and year as random effects. We found evidence for moderate brood-level effects on arrival time. Brood explained 12% of the variation in arrival time in the slope-only hierarchical model, whereas year explained only 2.7%. This suggests that annual movements out, and back into, Wytham Woods are generally consistent with at least some common brood-level causes impacting the timing of each individual.

### Competition for territory acquisition

Although parameter estimates for selection on individual phenotype and social selection were qualitatively similar in all three years, we found that effects in the second winter of study (2012/2013) were less clear-cut. However, in this second winter and spring, many fewer juvenile individuals were present in the population (152 vs. 520 and 411 juveniles in 2012 and 2014, respectively) potentially resulting in a reduction of competition for territories. By estimating local population size across the study area, we found that juvenile birds were competing against significantly more individuals (both adult and juvenile) per great tit nest box in the first and third years than the second year, whereas the third year did not differ from the first (see Table 3). Notably, competition in areas with a large local population (many individuals per nest box) led to an average competition that was much higher than the ratio of birds to nest boxes across the entirety of Wytham Woods (Year 1: 1053/1077 = 0.98, Year 2: 729/1077 = 0.68 and Year 3: 816/1077 = 0.76).

**Table 3:** Differences in estimated competition for territories each year. The second year (2012) had significantly fewer birds present, resulting in significantly lower competition. Significance levels calculated using an anova with a Tukey post hoc test

Year	Difference	95% range	*P*
2011/12–2012/13	0.345	0.489–0.203	< 0.001
2013/14–2012/13	0.282	0.128–0.438	< 0.001
2011/12–2013/14	0.063	0.205–0.079	0.554

## Discussion

We incorporated social network analysis into a multilevel analysis of selection, finding evidence that the social structure of phenotypes in a social network can mediate natural selection. We found that an individual's success in acquiring a territory was predicted not only by its own arrival time, but that the fitness cost of arriving late was also dependent on the arrival time of the conspecifics it associated with. Great tits were positively assorted by arrival time, resulting in areas with many early arrivers and others with many late arrivers. As a result, most late arrivals tended to associate with other late arrivals. Doing so had a positive effect on their chance of settling on a territory in the following spring compared to birds that arrived at the same time but into areas already well populated. Thus, the social component of selection could partially counteract strong negative selection on individual time of arrival. This clearly demonstrates how local interaction patterns can impact the overall selection operating on individual traits.

Our results revealed consistent directions and strengths of social and nonsocial selection gradients across three years. However, the slope estimate from the regression on the second year of data was not significant. This was likely due to reduced competition for nest boxes in 2013 compared to the other two years which was the result of poor breeding conditions in the spring of 2013 resulting in the lowest reproductive success recorded in the 53 year history of the full Wytham Woods study (Sheldon, B.C. unpublished data). Low breeding success led to a large reduction in the number of juveniles in the second winter, despite rather similar numbers of adults being present (533 in the first winter, 577 in the second, 411 in the third).

In order for social selection to occur, the social environment experienced by different individuals must vary. We found significant structure in the social network, with individuals assorting by arrival time. A recent study in this population found that assortment of birds in Wytham Woods into groups of immigrants and nonimmigrants is spatial (they occupy different areas) rather than social where they avoid flocking together in the same places (Farine, 2013b). Given the importance of prior residency on dominance in this species (Sandell & Smith, 1991), newly arrived individuals may be responding to competition by settling in areas of relative low population density rather than areas of high-quality habitat. This could be caused either by competition for food during the winter (important for both overwinter survival and body condition for breeding), or potential competition for good-quality territories when spring arrives. However, little is known about how individuals make decisions between options with different social conditions. Highly resolved temporal tracking of dispersing individuals in an intensively monitored population would be one way to establish how individuals trade-off different settlement options.

Social selection is likely to play an important role in mediating evolutionary dynamics in species that exhibit socio-behavioural flexibility (Silk *et al*., 2014). In fission–fusion societies, such as great tits (Aplin *et al*., 2012, 2013), groups can merge and split to form multilayered social structures that operate over a range of time scales (Couzin, 2006; Aplin *et al*., 2014; Farine *et al*., 2014). This flexible social structure is thought to facilitate behavioural plasticity as an adaptive response to changing environmental conditions (Lehmann & Boesch, 2004). However, it may also provide individuals with an opportunity to find a good social environment. In house finches (*Carpodacus mexicanus*), poorly ornamented males had a relatively greater probability of pairing with a female if they had greater social connectivity than poorly ornamented males with low social connectivity (Oh & Badyaev, 2010). By moving between groups, more social males were able to find an environment that increased their relative attractiveness, thereby reducing the selective load resulting from their own phenotype. If settlement decisions in great tits are driven by a strategy aimed at maximizing future breeding potential, then the social networks we observe are likely to be shaped by i) environmental conditions (the estimated breeding capacity of each site), ii) the arrival times of individuals and iii) the relative dominance rank of each individual.

Further, we found evidence for moderate brood-level effects on individual arrival times. This suggests that one factor that determined when individuals were first observed in the winter is the brood in which they fledged. This is likely to be primarily driven by birds from high-quality broods being disproportionately represented in the 33% of birds that remained in Wytham Woods over the summer. Although it does suggests that some cascading effects are likely to occur in this system (individuals that remain during summer have better territories, produce better quality chicks, and these also remain resident), the size of this effect is relatively small. For example, brood-level effects account for approximately 40% of fledgling mass (Garant *et al*., 2004), and 25% of the selection on fledging mass via first-year survival arised due to brood-level effects correlated with fledging mass (Bouwhuis *et al*., 2015).

One challenging concept for social selection is that individuals that gain benefits from their social environment through their social associations typically do so at the expense of their social partners. This is conceptually similar to Hamilton's model of the selfish herd (Hamilton, 1971), in which individuals reduce their relative risk of predation by moving to safer parts of the group, subsequently increasing the risk to others (maintaining an average 1/*N* risk across the herd). For example, Formica *et al*. (2011) found that in response to selection for large body size, male forked fungus beetles (*Bolitotherus cornutus*) could partially counteract this selection by associating with smaller conspecifics and hence transferring fitness costs to these. A further example in which individuals can benefit from disassortment in a frequency-dependent context is producer–scrounger games (Dubois *et al*., 2012). However, some cases may also exist where mixed-phenotype groups may generate benefits for all participants. Groups of great tits with different personalities may exhibit short-term emergent properties of collective behaviour that are not found in more uniform groups (Aplin *et al*., 2014). Similarly, in terms of predation, the rapid movement of phenotypically identical individuals can make tracking of prey more difficult for predators, thus impacting the probability that a given attack is successful (Landeau & Terborgh, 1986). As a result, the average predation risk is reduced to *a*/*N*, where *a* < 1. These examples highlight how population phenotypic structure in social animals can have a profound impact on selection. Failure to consider this constitutes a substantial omission in our ability to understand the process of natural selection.

This study provides evidence that selection on a phenotypic trait may be altered by patterns of interaction among individuals. Juvenile great tits were more likely to acquire territories if they arrived early, but late arrivals could increase their likelihood of breeding if they associated with other late arrivals. Importantly, this finding provides a evolutionary mechanism that could underpin patterns of social structure found in previous studies on this population, such as the spatial disaggregation of immigrant birds (Farine, 2013b). Given the growing number of studies gathering long-term social network data in animal populations (Farine *et al*., 2012; Rutz *et al*., 2012; Ryder *et al*., 2012), the ability to incorporate a network assortativity measure into models of selection will help us to elucidate the contribution of interacting phenotypes in social evolution.
